# Past and future of an IMI-PharmaTrain (IMI-PhT)-initiated multinational pharmaceutical medicine course at the Semmelweis University in Hungary

**DOI:** 10.3389/fphar.2024.1394987

**Published:** 2024-04-30

**Authors:** S. Kerpel-Fronius, M. Gottwald, P. Arányi, G. Renczes, A. Görbe, R. Papp, P. Ferdinandy

**Affiliations:** ^1^ Department of Pharmacology and Pharmacotherapy, Semmelweis University, Budapest, Hungary; ^2^ Independent Consultant, Berlin, Germany; ^3^ Ethics Committee for Clinical Pharmacology, Medical Research Council, Budapest, Hungary; ^4^ Retired, Budapest, Hungary; ^5^ Pharmahungary Group, Szeged, Hungary

**Keywords:** pharmaceutical medicine, medicines development course, PharmaTrain, Innovative Medicines Initiative, Cooperative European Medicines Development Course, university education, Central and Eastern Europe

## Abstract

The pharmaceutical medicine course at the Semmelweis University of Budapest, Hungary, was initiated as part of the Innovative Medicines Initiative (IMI is the main program, IMI-PharmaTrain is one of the IMI projects) Pharmaceutical Medicine Training Programs (16 IMI Call 2008/1/16). The aim was to extend training in the development of pharmaceutical medicine to those EU member states where no such education was present. The final program envisaged the development of a cooperative education supported by universities located in Central and Eastern Europe. It was considered to be the economically and scientifically most viable approach to combine the expertise from these countries to form a united teaching staff and provide education jointly for young professionals of the region. Semmelweis University was selected to manage this coordinated program. In this report, we describe the organization and functioning of this international university-based pharmaceutical medicine education project called the Cooperative European Medicines Development Course (CEMDC) and evaluate its successes and shortcomings. During the pandemic, the educational course was interrupted. The follow-on program is reorganized as a postgraduate MSc course named “Semmelweis Pharma MBA” and will be started in 2025. It will continue the established PharmaTrain educational tradition. However, it will deal in more detail with the transition from basic pharmacological to industrial research, as well as biopharmaceutical formulation and manufacturing and marketing aspects of medicines development.

## Introduction

The very rapid increase of the scientific knowledge underpinning modern medicines development presents a great challenge for both pharmaceutical and medical device industries as well as for the healthcare system. Intensive cooperation between industry experts, treating physicians, and recently, non-medically trained scientists has become a well-accepted approach to develop highly complex advanced therapies. Already, in the second half of the 20th century, the need to provide complex training for experts working in both pharmaceutical and healthcare industries has become apparent. This has led to the emergence of pharmaceutical medicine as a new medical specialty since earlier only medically trained personnel were engaged in clinical development, both in the pharmaceutical industry and in hospitals. The professional organization of medical advisers working in the pharmaceutical industry was started in the 1970s (Stonier et al., 2007). In 1975, 12 national pharmaceutical medicine societies formed the International Federation of Associations of Pharmaceutical Physicians and Pharmaceutical Medicine (IFAPP) (Stonier et al., 2007; IFAPP). According to the generally accepted definition, “Pharmaceutical Medicine is the medical scientific discipline concerned with the discovery, development, evaluation, registration, monitoring and medical aspects of marketing of medicines for the benefit of patients and public health.” (IFAPP).

Pharmaceutical medicine courses were offered first in countries having large, worldwide-acting pharmaceutical industries. In regions having smaller pharmaceutical companies predominantly producing generic drugs with a low profit margin, such training was mostly absent due to the lack of money available for funding the education of employees. However, with the increasing local production of medicines for supporting national healthcare, it has become necessary to train experts in pharmaceutical medicine in many countries with moderate and/or developing pharmaceutical industries. Such expertise became also necessary for physicians organizing international and local clinical trials. Parallel with the rapidly increasing number of non-medically trained specialists involved in complex therapies, the course material also had to be adapted to cover the needs of this new group.

The vision to improve the teaching of medicine development in the EU led to the Innovative Medicines Initiative PharmaTrain (IMI-PhT) project. Its aim was to promote the cooperation between academic centers and pharmaceutical companies for modernizing and harmonizing the education of medicine development courses offered by various universities and to extend professional education to new EU member states in Central and Eastern Europe (CEE).

## The organization of the IMI-PhT program and the foundation of the CEMDC

The IMI was a project of the European Union implemented in 2008. It is a public–private partnership between the European Union (represented by the European Commission) and the European pharmaceutical industry (represented by the European Federation of Pharmaceutical Industries and Associations, EFPIA). The aim of this partnership was to modernize and speed up medicine development and to improve patient access to innovative medicines in general and in particular in areas of unmet medical needs. To achieve this goal, the IMI facilitated intense collaboration between universities, research centers, patient organizations, regulatory bodies, and the pharmaceutical industry (IMI). The 5-year-long IMI-PhT cooperation was started in 2009 in the first round of accepted IMI projects (PharmaTrain a). First, the project was called the European Federation of Course Providers in Pharmaceutical Medicine (EFCPM). It was renamed to PharmaTrain in 2012. Altogether, 15 pharmaceutical companies representing the EFPIA and 33 universities, research organizations, public bodies, and non-profit groups, including the Semmelweis University of Hungary, participated in the IMI-PhT project. The main achievement of the PhT cooperation was to provide shared standards and guidelines for postgraduate pharmaceutical medicine courses. This was realized by updating the teaching topics listed in the IFAPP syllabus (The PharmaTrain Syllabus, 2018) and defining the expected learning outcomes of the education. It also suggested precise criteria for the evaluation of medicine development courses (IFAPP World, 2015). The PhT organized a network of 13 courses recognized as Centers of Excellence because of fulfilling all the agreed educational standards. One of them was the CEMDC (Cooperative European Medicines Development Course), which was established during the lifetime of the IMI-PhT program. After the termination of the IMI-PhT project in 2014, the cooperation of centers teaching the PhT program continued in an independent follow-up organization called PharmaTrain Federation (PharmaTrain b).

One of the aims of the PhT program was to establish a pharmaceutical medicine course in CEE, where education on medicine development was not yet well-developed. It was known that local courses were already organized earlier in the Czech Republic and the Republic of Serbia; however, these countries did not participate in the PhT project. On the other hand, Hungary joined the IMI-PharmaTrain program because a pharmaceutical medicine course was already initiated at Semmelweis University in Budapest using the available IFAPP educational guidelines. In addition, Hungary has a relatively well-developed pharmaceutical industry firmly established in the region. Considering these circumstances, the participants selected the Hungarian course for further support in the PhT project. Semmelweis University accepted this challenge and volunteered to modify its already running course according to the new ideas emerging during the PhT discussions (Kerpel-Fronius, 2015). Two persons were selected to manage this program: Matthias Gottwald of Bayer, representing the EFPIA, and Sandor Kerpel-Fronius, serving as the study director of the running Hungarian course.

The geographical extension plan envisaged supporting one course in CEE to work according to the PhT standards. However, this approach would not solve the issue of education of experts working in the other countries of CEE. Therefore, following the suggestion provided by Hungary, we began to think of a course jointly run by several universities located in CEE. After a thorough discussion and with the energetic support of Professor Fritz Bühler, head of the IMI-PhT project, a draft plan was produced. It envisaged organizing a cooperative course having its administrative center at Semmelweis University in Budapest. The idea was to involve the teaching staff from the universities located in CEE countries for offering pharmaceutical medicine development education jointly to an entire region. We realized that none of these countries have enough experts and students for running individual courses efficiently. The draft plan was accepted both by the IMI program and by Semmelweis University, which also agreed to manage the administrative work associated with international education.

**FIGURE 1:**
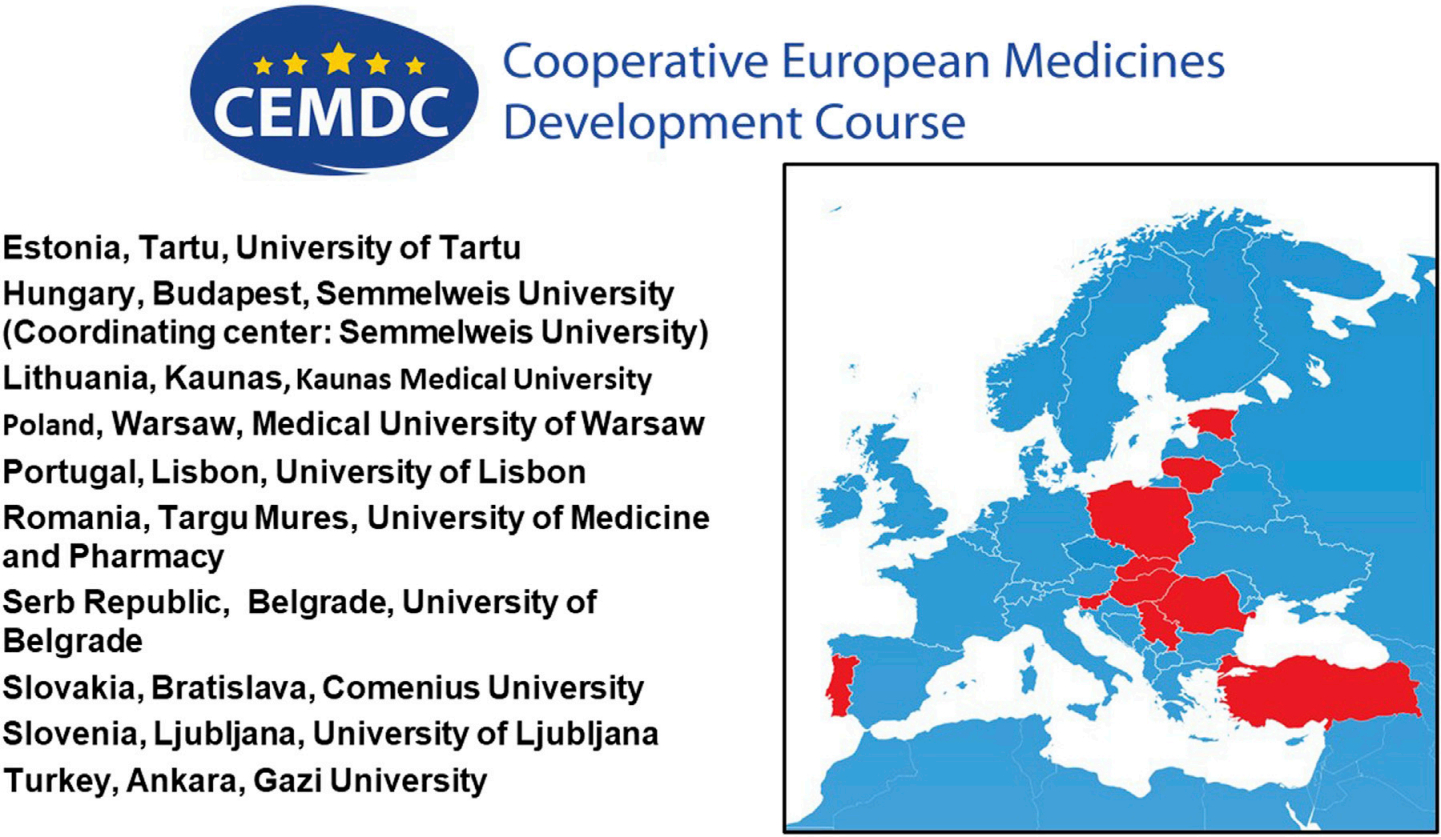
Participating countries and universities in the CEMDC.

Following the principal agreement on the organization of the CEMDC, many universities were contacted. Originally, 16 universities signaled interest, some of them located outside CEE. The PhT generously sponsored five joint meetings at which the interested universities discussed many administrative and practical aspects of joint education. It was decided that all administrative issues of all enrolled students will be handled, according to the Hungarian Law, by Semmelweis University. A governing board representing all participating universities will define the actual course program based on the PhT principles and syllabus. They will also elect a study director responsible for managing all educational and administrative issues during the course. Finally, Semmelweis University was appointed to provide quality control over the overall management of education, while the quality control over the pharmaceutical medicine-teaching program remained the responsibility of PhT, similar to all other courses. Based on these discussions, relevant standard operating procedures were prepared. The rectors of 10 participating universities finally signed the Memorandum of Understanding at the inauguration ceremony of the CEMDC, which took place in Budapest at Semmelweis University on 30 October 2012 (Figure 1).

As mentioned above, the first Hungarian pharmaceutical medicine course was already running at Semmelweis University in 2009 when the PhT project was initiated. We used the six basic modules of the IFAPP pharmaceutical medicine course program together with the IFAPP syllabus. These were further developed during the IMI program and renamed to the PhT course and PhT syllabus. Therefore, it was relatively easy to adapt the new modifications to our running program given in English. Many participants of the PhT cooperation accepted kindly to lecture at the Hungarian course and also supported us by providing guidance regarding the best practices for course management, examination practices, and quality control applied in IFAPP courses.

One of the most important goals of the PhT cooperation was to develop a master level program by adding six freely selectable elective modules to the basic course. Since we also planned to develop the CEMDC to become later a master course, we decided to add several topics of the newly developed elective modules to the running program. These were primarily dealing with health economics, biological and advanced therapies, pharmacovigilance, and follow-on generic and biosimilar medicinal agents. Semmelweis University together with experts from the Hungarian pharmaceutical industry prepared this last module. Finally, the continuously adapted first course contained 319 teaching hours, of which 179 and 140 belonged to the base and elective modules, respectively. Twenty-six students started the first course, of whom 22 finished the basic course and 20 also completed the master-level modules and defended a thesis. Of those who followed the entire course, 10 were physicians, 5 were pharmacists, and 5 had MSc degrees in natural sciences. Essentially, during this IMI-PhT training phase, almost a full program for a master course was developed. This first course was quality-controlled by PhT in 2013. They concluded that the Hungarian course reached the PhT quality standards and recommended to award the title “PharmaTrain Center of Excellence.” They also reviewed the organization plan for the new international cooperative course and found it satisfactory for initiating the joint CEMDC education program (Report of the PharmaTrain Assessment Report, 2013; Kerpel-Fronius, 2015).

## The learning environment and the educational program of the CEMDC between 2013 and 2019

In this section, the experience obtained in three consecutive CEMDC courses provided between 2013 and 2019 is presented jointly. Unfortunately, the great expectations of the PhT geographical extension program could be fulfilled only partially because the international and local pharmaceutic companies operating in CEE failed to support their coworkers to participate in the course. Frequent traveling because of face-to-face teaching also limited international enrollment. Indeed, in addition to the Hungarian participants, only two students registered from Lithuania followed and finished the CEMDC course. This was not only a local problem. Just when finishing the IMI-PhT project, the pharmaceutical industry began to withdraw from the joint program claiming that the combined basic-master course is far too expensive and time-consuming for the pharmaceutical industry. Instead, the companies reverted to their internal training model providing limited practice-oriented education for their employees and short external training courses on specific topics. In addition, budgets for training were primarily linked to central units, while the subsidiaries in CEE countries mostly lacked sufficient budgets for such programs. As the headquarters are almost all located in Western Europe, they usually sent their employees to training centers in their regional vicinity.

The original organizational plan of the CEMDC had to be modified because of the low number of students registering for the course. Nevertheless, we considered continuing the CEMDC joint program supported so generously by the IMI-PhT cooperation as an ethical obligation. In addition, several Hungarian and a few foreign students already registered for the course, covering privately their tuition fees. In the following three courses, we had 8, 6, and 7 students finishing the entire course. Of the students, 29% were physicians, 26% pharmacists, and 39% natural scientists. We accepted also one lawyer and one economist because of their extensive working experience in medicine development. In addition, several students from different European courses participated in selected modules since according to the PhT agreement, the modules finished in courses following the PhT program were mutually recognized. We managed the program exclusively with the participation fee of the students. It was a great support that Semmelweis University offered its facilities and administrative background free of charge. In addition, many colleagues did not ask for honoraria, and we had to support only their travel and local expenses.

Our main goal was to maintain the international university network concept at least in a modified form primarily based on email communication with university representatives. In practice, the study director working at Semmelweis University planned, executed, and supervised the courses. He prepared and distributed the advertisement material and the draft program to the participating countries. The national representatives were asked to advertise the program locally, provide their comments, and nominate possible local lecturers for the planned modules. In addition, for supporting the international cooperative concept of the CEMDC, professors Mlinaric-Rascan and Klimas offered to organize modules 2 and 5 in Slovenia and Slovakia, respectively. This provided the opportunity to involve many local experts as lecturers. In Slovenia, additional students joined the core pharmaceutical medicine audience since the module dealing with non-clinical and early clinical development was included in their local PhD program. These students received the standard modular certificate issued by Semmelweis University after passing the examination at the end of the module. However, they were not accounted as pharmaceutical medicine course participants. Following the Slovenian example, the PhT modules were later accepted by the Semmelweis University PhD program as freely selectable topics. This linking between different programs was a unique and very fruitful cooperation facilitated by teaching pharmaceutical medicine in the university environment.

Altogether, 103 lecturers participated in the three consecutive courses. The largest proportion (36%) of the lecturers came from academic institutions. The industry, the contract research organizations, and the regulatory agencies contributed 29%, 16%, and 19% of the teachers, respectively. Of the lecturers, 45% were Hungarian. Several foreign lecturers were former participants of the IMI-PhT cooperation, who kindly offered their continuous support, while other experts were recommended by the participating universities of the CEMDC project. If needed, we invited also lecturers with special expertise without any formal ties with the PhT program.

The plan to provide a complete MSc course also was modified primarily due to the limited financial resources of the students to cover the entire program. The second course only contained the six base modules. Later, the program was extended since we considered it important to present new developments in pharmaceutical medicine. Pharmacoeconomy was included in the revised basic Module 6 dealing with healthcare economics. In addition, two new elective modules, one covering biologicals and advanced medicinal products and another on follow-on generic and biosimilar agents, were added. This extension also satisfied the criteria for retaining the PhT Center of Excellence qualification. Topics dealing with ethics were presented in Module 1 and were additionally discussed in more detail in a case discussion dealing with trial organization. In all modules, much time was devoted to carefully explain the relevant scientific background of the topics (Table 1).

**TABLE 1:** Titles and teaching hours of modules (M) presented in courses (C) 2, 3, and 4.

M #	Module titles	C2	C3	C4
M1a	Introductory program: an overview	5	6	10
M1b	Principles of discovery of medicines and development planning	18	19	14
M2	Non-clinical, pharmaceutical, and early clinical development	24	25	24
M3	Clinical development of medicines: exploratory and confirmatory	24	25	26
M4	Clinical trials	24	22	23
M5	Regulatory affairs; drug safety and pharmacovigilance	25	34	25
M6	Healthcare marketplace: economics of healthcare	25	28	31
M7	Biological medicinal products and advanced therapies	--	24	24
M8	Follow-on drugs: generic, biosimilar, and non-biological similar medicinal products	--	25	24
	**Percentage of hours devoted to workshops and case discussions per course**			
	Workshops	6.2%	5.2%	8.5%
	Group discussions	20.6%	14.4%	7%
**Total**		**145**	**208**	**201**

## Pedagogical principles and quality standards underlying the CEMDC educational activity

### Learning outcomes (Los)

The PhT program provides a competency-oriented education. For supporting this pedagogical goal, the experts writing the modules have to define specific learning outcomes (LO), which have to be achieved by the education program. Usually, 8 to 10 LOs are defined per module. In the programs prepared for a given module, the relations of the lectures both with the respective topics listed in the PhT syllabus and with the predefined LOs are indicated. In our course, the study director was responsible to guide the lecturers in preparing their material in harmony with these requirements. The firm link of LOs with the program was further emphasized by asking the students to rate how far the specified learning outcomes (LOs) were supported by the lectures. In addition, the quality of the lectures was rated according to expectation, content, and presentation.

### MCQ examination

Each module was followed by an MCQ (multiple-choice questionnaire) examination with around 30 MCQs. Many MCQs were received from the central IMI-PhT pool, while others were prepared by the study director in cooperation with the lecturers as required. The “multiple true–false questions” (MFT) format of MCQs was used. The MCQs consisted of a question or a statement followed by five suggested answers. Any number of the suggested answers could be correct or false since the answers were not related. The students had to mark each proposed answer separately to be true (T) or false (F). One credit was given if all five of the proposed answers were correctly evaluated to be T or F; 0.75 and 0.5 credits were gained by providing four or three correct answers. No credit was obtained if only two or less answers were correct since in such cases, it was obvious that the student could not evaluate the scientific content of the question. The summary marking of the MCQ test according to the requirements of Semmelweis University from 5 to 1 was based on the % of the points obtained. We calculated the maximum number of points achievable by answering all MCQs correctly. The highest note of 5 was given if the student obtained 100%–90% of the maximum credits. The other notes were related to the following percentage of good answers: 4 (89%–75%), 3 (74%–60%), and 2 (59%–50%). Students failed if they achieved <50% of the points. Students who did not pass could repeat the examination by answering the same MCQs.

This MCQ format using five true or false answers was unknown to the students. During the first few modules, several students failed; however, the results improved significantly by gaining experience with this MCQ examination. The quality check of the MCQs was performed together with the students. After each examination, we projected the MCQs and explained the correct answers. If the joint discussion proved that one or two answers to an MCQ were not correctly formulated, we rewrote the answers for future use of the MCQ. Our experience indicated that this type of MCQ correctly represented the knowledge of the students and made the scoring system more reliable than the conventional “single best answer question.” This impression is in line with observations of Brassil and Couch, who found in a comparative study that the MTF type of MCQ better characterizes the understanding of the addressed complex problems by the students than the more frequently applied single-best answer multiple-choice (MC) format (Brassil and Couch, 2019). The confidentiality of the MCQs was guarded very carefully.

### Short assignments

After each module, the students had to submit a formative essay on the same subject selected by the teachers. Open-access papers related to the subject were provided. The goal was to educate students in writing short, concise reports and correctly citing the literature. The length of the assignment was defined between 800 and 1,200 words; shorter or longer assignments were considered a mistake. The short essays were evaluated using an assignment evaluation form using the format introduced during the PhT program by King’s College. The scoring was performed according to three to five predefined criteria per section. The three main sections of the form covered the selection and coverage of the material, the understanding of the issues, and finally, the structure of the presentation. The sum of maximal points was 60. The final grade was based on the % of points reached (excellent, grade 5 [60–54 points; 100%–90%]; failed, grade 1 [<30 points; <49%]). Comments given by the evaluators helped the students improve in writing short reports. For providing pedagogical consistency, the assignments were evaluated and commented on by the same two evaluators throughout the entire course.

### Final examination and writing a thesis

The final examination was based on answering around 100 MCQs, and the students had to prepare a thesis based on topics selected by them. The only restrictions were that it should be connected to the non-clinical or clinical development of medicinal products and that the study director had to agree to the recommended subject. The aim was to train the students in searching and critically interpreting data of the scientific literature. The length had to be between 50,000 and 100,000 characters. The thesis was evaluated by the same method used for the essays, however, in this case by three examiners. The summary of the thesis had to be presented at the final oral examination in a 20-min-long free lecture, followed by questions related to the thesis and the entire course material. The examination board consisted of three members. One had a professional background in industrial drug research, while another in clinical drug evaluation. The third member was the study director. Of the three evaluators, two asked questions and one functioned as a notary of the examination. The mean score of the MCQs, the thesis, and the oral examination was calculated to provide the final grade.

### Quality control

Semmelweis University registered the CEMDC course at the Hungarian Educational Board as a continuous professional development program. All the data related to the course and the academic records of the students were entered into the NEPTUN electronic database, which is an online educational administration system for students enrolled in Hungarian universities. On this basis, Semmelweis University issued the degree certificate in English. We also provided a description of the modules given according to the terminology used by PhT.

## Reorganization and modernization of the future course

In 2019, after finishing the fourth CEMDC course, a pre-planned generation change in the leadership of the course was made due to the retirement of several key participants, including the study director. Unfortunately, due to the COVID-19 pandemic, the reorganization was delayed. The new course in the form of a postgraduate MSc course is planned to start at Semmelweis University in 2025. The rapid progress in medicine and medical device development together with the broad introduction of Internet-based distance teaching necessitates rethinking both the program and the organization of the education course. Considering the very low number of students who registered from the participating countries, the continuation of the joint CEMDC educational system is not considered practicable. Accordingly, Semmelweis University will manage the course alone in the future. Nevertheless, leading experts from other international universities and the pharma industry will be involved. The course will be open to foreign students, and the language of education will remain English.

The new postgraduate MSc course ‘Master in Pharmaceutical Innovations and Business Administration,’ or shortly ‘Semmelweis Pharma MBA,’ will continue the PhT educational traditions and will remain firmly anchored in the university teaching environment, but industrial experts will play a more dominant role in shaping the program. The primary aim of the new educational concept will be to more closely integrate basic pharmacological research with innovations in medicine development. It will incorporate the topics listed in the PhT syllabus but will deal in more detail with the transition from basic research to pharmaceutical development, patent protection of new molecules emerging from university research, and management of spin-off companies. Pharmaceutical formulation, manufacturing of medicines, management of supply chains, business administration, marketing, pricing, and reimbursement will play a prominent part as well. The development of medical devices and the use of AI in medicinal research will also receive more attention. The scientific topics will be presented and associated more closely with related production and marketing issues. Distant and face-to-face education will be combined to make participation easier.

## Discussion

The IMI-PhT project was a significant milestone in developing pharmaceutical medicine as an independent medical–scientific specialty. For non-medically trained students, the program planned to provide a master’s degree in science. The recommended educational program reflected many accepted principles of training medical specialists as well as other scientists working in academic research. Retrospectively, this academic focus might have been one of the causes that the pharmaceutical industry did not enthusiastically support the outcome, and many companies returned to their internal training programs, mostly limiting the teaching to the specialized tasks the attendants should be able to perform in the industry. As a result, the expected increased influx of students from the industry to academic pharmaceutic medicine training courses did not happen. The lacking enthusiasm in the industry also led to the limited success of the geographical extension program of the PhT cooperation. Only very few students were registered by the international and local companies for the long, complex, and expensive training. Several students covered the education cost, at least partly, from their own pocket.

The envisaged multinational, cooperative academic educational concept was a revolutionary new idea. It was a very courageous decision both by the leaders of the PhT cooperation and by the IMI program coordinators to enthusiastically accept and support this bold plan. No such experience was known in the European educational environment. In the existing international university cooperations, usually, two partners educate different parts of a combined curriculum separately. The participating universities issue separate national qualifications after finishing their section of the program, which together certify the completion of the education course. The aim of the Lisbon Convention was “to facilitate the recognition of qualifications granted in one Party in another Party,” which provided the legal background of this type of university cooperation (Lisbon Recognition Convention (Council of Europe)). The problem with the recognition of a joint PhT degree of the CEMDC was that it did not formally belong to any national education system and consequently was not covered by the Lisbon Recognition Convention. According to the final agreement reached after prolonged negotiations with the support of PhT and the International Department of Semmelweis University, the participating universities entitled Semmelweis University to enroll all the students and issue their qualifications. This solved the problem and demonstrated the real cooperative spirit of the participants. Although only few foreign students participated, in the case of two students from Lithuania who finished the entire course, we demonstrated that this new organizational system worked. A modular certificate issued by Semmelweis University also effectively supported the mobility of the students since these modular qualifications were mutually acknowledged by the courses following the PhT curriculum. This made possible that some students from other courses came to follow modules in Hungary, and Hungarian students could continue their studies for a master’s degree at another PhT course. Finally, the University of Ljubljana accepted the certificate of Module 2 for their PhD students.

An important achievement of the CEMDC was the introduction of the improved PhT educational standards in CEE, which were well-accepted by both the teachers and the students. The submission of short essays after each module was very useful for teaching to write short and informative comments on a given subject. The acquired teaching experience will be helpful to continue pharmaceutical medicine education in the PhT tradition at Semmelweis University. The close cooperation of the university and the pharma industry in planning the new program and the extension of the PhT program to include more industry-relevant topics is expected to improve academic–industrial cooperation. We hope that these changes will encourage the pharmaceutical industry to enroll more students. This would be necessary since many industry experts consider broad continuous professional education in pharmaceutical medicine to be crucial for the future of medicine development (Imamura et al., 2019).

Although the geographical extension of the PhT program did not fully reach its goal to educate many regional students, mostly because of the few students enrolled by the pharmaceutical industry, its educational success should not be underestimated. It developed a unique international university cooperation to jointly educate students from the region. Although the number of non-Hungarian students finishing the course was low, it demonstrated that this bold, new type of international university cooperation is functional. The system might be operated more easily in the future using the technology of distant teaching. For Hungary, the CEMDC educated a new generation of medicine development experts, and some of them reached leading positions. They were also the same people who later organized the Pharmaceutical Medicine Section of the Hungarian Society of Experimental and Clinical Pharmacology (huphar.org). They will also contribute to pharmaceutical medicine education in the “Semmelweis Pharma MBA” starting in 2025.
